# ORIF of three- and four-part proximal humeral fractures: a retrospective study of long-term clinical and complication outcomes of two-plate fixation methods

**DOI:** 10.1186/s10195-026-00914-w

**Published:** 2026-03-15

**Authors:** Jacopo Conteduca, Elena Gasbarra, Igor Rausa, Alberto Casto, Luigi Valentino, Giuseppe Solarino, Luigi Meccariello, Giuseppe Rollo

**Affiliations:** 1 Department of Clinical Sciences and Translational Medicine, Tor Vergata University of Rome, Rome, Italy; 2 Department of Orthopaedic and Traumatology, Vito Fazzi Hospital, Lecce, Italy; 3 Department of Translational Biomedicine and Neuroscience, Università Degli Studi Di Bari Aldo Moro, Bari, Italy; 4 Department of Orthopedics and Traumatology, AORN San Pio Hospital, Benevento, Italy

**Keywords:** Long-term outcomes, ORIF, Plate, Proximal humeral fracture

## Abstract

**Background:**

The aim of this study was to compare clinical outcomes and complication rates during long-term follow-up (≥ 6 years) after treatment for three- and four-part proximal humeral fractures using two different types of locking plates.

**Materials and methods:**

A total of 113 patients with three- and four-part proximal humeral fractures who underwent surgery between September 2012 and January 2019 were enrolled retrospectively.

Data for 49 patients [ 9 males, 40 females; mean age [standard deviation] (SD ) 68.9 (5.8) years} treated with a PGR (intrauma) plate (group A) and 51 patients [10 males, 41 females; mean age (SD): 66.0 (7.5) years] treated with a PHILOS humeral plate (PHP, group B) were available at the last follow-up. The mean follow-up periods in groups A and B were 10.8 and 10.2 years, respectively, with a minimum follow-up of 6 years. At the final follow-up evaluation, functional outcomes were assessed using the Oxford Shoulder Scale (OSS), Simple Shoulder Test (SST), and Disabilities of the Arm, Shoulder and Hand (DASH) questionnaires. X-ray evaluation was also performed, and complications were recorded.

**Results:**

The mean OSS score in the PGR and PHP groups at follow-up was 42.9 and 39.1, respectively (*p* > 0.05). The SST score (PGR: 7.5 ± 2.0; PHP: 7.3 ± 4.0) and DASH score (PGR: 22.1 ± 5.5; PHP: 20.5 ± 4.0) were similar in both groups (*p* > 0.05). In our series, 6% of patients had complications; these included avascular necrosis of the humeral head (two cases in the PGR group and three in the PHF group) and one case of periplate fracture in the PGR group.

**Conclusions:**

Among patients with three- and four-part proximal humeral fractures, both PGR plate and PHP treatment achieved comparable functional outcomes and demonstrated similarly low complication profiles. These results reinforce the appropriate clinical utility of either implant for fixation in the treatment of this fracture pattern.

Level of evidence: 3

## Introduction

Proximal humeral fractures (PHFs) are common injuries and continue to pose a therapeutic challenge in clinical practice [1]. They most frequently occur as fragility fractures in elderly patients after low-energy trauma, while high-energy mechanisms such as falls from height or sports injuries are more typical in younger individuals [2]. A variety of therapeutic strategies exist for the management of PHFs, with the most frequently employed being conservative immobilization in a sling, open reduction and internal fixation (ORIF), intramedullary nailing, and shoulder arthroplasty. The determination of the optimal treatment modality remains controversial and is typically guided by patient-specific characteristics and the clinical judgment of the treating surgeon [3].

ORIF with locking plate fixation is a conventional approach for proximal humeral fractures [4, 5]. Compared with blade plates, locking plates offer benefits such as high elastic stiffness, strong fatigue resistance under axial compression, and greater stiffness during cyclical external rotation [6].

In addition to conventional open reduction and internal fixation, minimally invasive plate osteosynthesis (MIPO) has been proposed for the treatment of proximal humeral fractures, particularly with anatomically contoured locking plates such as PHILOS and other systems available on the market. MIPO aims to preserve soft tissues and vascularity, potentially reducing surgical trauma; however, its indications and long-term outcomes in complex fracture patterns remain debated [7].

However, locking plate fixation is frequently associated with substantial rates of complications, including loss of reduction and screw penetration [8]. Both biomechanical and clinical studies have explored the use of bone cement to augment either the humeral head or the locking screws, aiming to improve the initial stability of the bone–implant construct [9].

Moreover, the most appropriate implant type has not already been defined, although the Proximal Humerus Internal Locking System (PHILOS), an interlocking anatomically recontoured plate, is the most commonly investigated plate [10].

The patellar groove replacement (PGR) plate (Intrauma s.p.a.) has previously been compared with the PHILOS plate, showing equivalent short-term outcomes [11]; however, long-term outcomes have never been investigated.

The aim of this retrospective study was to compare the clinical outcomes and complications of ORIF with a PGR plate or ORIF with a PHILOS humeral plate (PHP) for three- and four-part fractures of the proximal humerus after a minimum follow-up of 6 years.

## Materials and methods

This nonrandomized retrospective study was approved by the local institutional review board (IRB)/research ethics committee (approval no. 1875 CEL) and performed in accordance with the ethical standards laid down in the 1964 Declaration of Helsinki.

Between September 2012 and January 2019, we identified 191 patients from our surgical database who had sustained a proximal humeral head fracture and were surgically treated at a single center (Vito Fazzi Hospital, Lecce, Italy). The inclusion criterion was the presence of an acute (treatment within 15 days from trauma) type III or IV fracture pattern according to the Neer classification [12]. The exclusion criteria were split head fracture pattern or humeral head luxation, open fracture, bilateral fractures, pathological fracture, and previous ipsilateral upper limb surgery. Patients were excluded if they had neurological disorders, cognitive impairment, or medical conditions that precluded reliable completion of the functional questionnaires or attendance at follow-up visits. On chart review, 78 patients were found not to meet the inclusion criteria. Of the 113 remaining patients, 3 refused to return for clinical evaluation or were lost to follow-up, 8 had died from unrelated causes, and 2 had senile dementia that prevented them from completing the required questionnaires.

The choice between the PGR plate and the PHILOS plate was based on the operating surgeon’s preference and implant availability at the time of surgery.

The final study groups included 49 patients treated with a PGR plate (Intrauma s.p.a.) (group A) and 51 patients treated with a PHP (Synthes DePuy GmbH, Oberdorf, Switzerland) (group B). Fractures were categorized using preoperative plain X-rays in combination with computed tomography scans.

All participants in both groups received clear and thorough information regarding the treatment approach, as well as other potential surgical and conservative options. The study followed the ethical principles outlined in the Declaration of Helsinki, and all patients were asked to read, understand, and sign the informed consent document.

### Surgical technique

All patients were placed in the beach-chair position, and a deltopectoral surgical approach was consistently utilized. After meticulous soft tissue dissection and optimal exposure of the fracture site, anatomical reduction of the fracture was achieved. To enhance construct stability, nonabsorbable sutures were placed through the insertion sites of the respective rotator cuff tendons.

Bone substitutes were selectively used to augment fixation in cases with disruption of the medial hinge to restore medial column support. This was required in three patients in group A and in two patients in group B. No autologous bone grafts were used.

Plate placement was confirmed before screw drilling, and the final construct was again checked fluoroscopically to ensure appropriate screw length and positioning. Postoperatively, the arm was immobilized in a sling for 4 weeks, and standardized rehabilitation was initiated on the first day after surgery.

### Clinical follow-up and evaluation

All postoperative assessments were conducted by an orthopedic trauma surgeon at our institution at a minimum follow-up time of 6 years. Any postoperative complications involving the proximal humerus were documented. Functional outcomes were evaluated using the Simple Shoulder Test (SST) scale [13], the Oxford Shoulder Score (OSS) questionnaire [14], and the Disabilities of the Arm, Shoulder and Hand (DASH) questionnaire [15].

Postoperative complications were recorded and defined as any adverse event involving the proximal humerus during follow-up. Specifically, complications included avascular necrosis of the humeral head, hardware-related complications, and events requiring additional surgical or medical treatment.

### Statistical analysis

Descriptive statistics were calculated for all variables, with means, standard deviations, medians, interquartile ranges (P25–P75), and minimum and maximum values reported for continuous variables and frequencies for categorical variables. Group comparisons of continuous variables (age and follow-up duration) were conducted using independent-samples *t*-tests, and Levene’s test was applied to assess the equality of variance. For categorical variables (Neer classification), Fisher’s exact test was used.

The two treatment groups were not homogeneous: Significant differences were observed in age, follow-up duration, and fracture-type distribution. To minimize these imbalances, propensity score matching (PSM) was performed using a logistic regression model that included age, follow-up duration, and Neer classification as covariates. Nearest-neighbor 1:1 matching without replacement and a caliper of 0.25 on the logit of the propensity score were applied, resulting in 44 matched pairs (88 patients total). After PSM, no significant differences remained between groups for age, follow-up, or fracture-type distribution, confirming balance.

After matching, functional outcomes (OSS, DASH, and SST scores) were compared between groups using independent-samples *t*-tests, and Levene’s test was used to verify variance equality. Cohen’s *d* was calculated to estimate effect sizes, and the results were evaluated against minimal clinically important difference (MCID) thresholds to assess clinical relevance [16–18].

Subgroup analyses by age (< 65 versus ≥ 65 years) were performed. Given the small sample sizes, normality could not be assumed; Shapiro–Wilk tests confirmed significant departures from normality (*p* < 0.05) for all outcomes. Consequently, Mann–Whitney *U* tests were used for between-group comparisons within age strata.

Complication incidences were compared between groups using Pearson’s chi-squared test for proportionality. All analyses were conducted with IBM SPSS Statistics version 29 and R (MatchIt package).

## Results

### Patient population and PSM efficiency

The descriptive statistics for the whole sample are presented in Table 1. The initial cohort showed significant disparities: PHILOS patients were older (*p* = 0.033), had longer follow-up (*p* = 0.015), and had fewer Neer type 4 fractures (*p* = 0.025) compared with the PRG group (Tables 2 and 3). PSM effectively eliminated these differences, resulting in 44 matched pairs (88 patients total) with balanced baseline characteristics (Table 4). Specifically, after matching, age (*p* = 0.155), follow-up duration (*p* = 0.070), and Neer fracture distribution (*p* = 0.132) were statistically homogeneous (Tables 5 and 6).

**Table 1:** Descriptive statistics for the entire sample

	Count	Percentage (%)	Mean	Standard deviation (SD)
Group				
A: PGR	52	52.0%		
B: PHILOS	48	48.0%		
Neer classification				
3	72	72.0%		
4	28	28.0%		
Complications				
Absent	95	95.0%		
Necrosis	5	5.0%		
Follow-up (years)			7	2
OSS (Oxford) score			40	10
DASH score			16.542	21.190
SST score			10.02	2.54

**Table 2:** Independent *t*-test

	Group	*N*	Mean	SD	*t* (df)	*p*-Value
Age (years)	A: PGR	52	65.96	10.324	−2.167 (97.1)	0.033
	B: PHILOS	48	70.48	10.500		
Follow-up (years)	A: PGR	52	7.06	1.290	−2.485 (79.7)	0.015
	B: PHILOS	48	7.90	1.981		

*df* degrees of freedom for pre-matching comparisons

**Table 3:** Fisher exact test for pre-matching comparisons

	Group		Total	
	PHILOS	PRG		*p*-Value
Neer classification				
3				
Count	40	32	72	0.025
Percentage (%) within Neer classification	55.6%	44.4%	100.0%	
Percentage (%) within group	83.3%	61.5%	72.0%	
4				
Count	8	20	28	
Percentage (%) within Neer classification	28.6%	71.4%	100.0%	
Percentage (%) within group	16.7%	38.5%	28.0%	
Total				
Count	48	52	100	
Percentage (%) within Neer classification	48.0%	52.0%	100.0%	
Percentage (%) within group	100.0%	100.0%	100.0%

**Table 4:** Variables before and after propensity score matching

Variable	Mean group A (before)	Mean group B (before)	Mean group A (after)	Mean group B (after)
Age (years)	65.9	70.5	67.5	70.5
Follow-up (years)	7.1	7.9	7.1	7.8
Neer type 3 (%)	61.5	83.3	68.2	84.1
Neer type 4 (%)	38.5	16.7	31.8	15.9

**Table 5:** Independent *t*-test

	Group	*N*	Mean	SD	*t* (df)	*p*-Value
Age	PRG	44	67,522	8927	−1.435 (86)	0.155
	PHILOS	44	70,500	10,475		
Age at the follow-up time	PRG	44	7136	1268	−1.842 (72.6)	0.07
	PHILOS	44	7795	2006		

*df* degrees of freedom after matching

**Table 6:** Fisher exact test for Neer classification after matching

	Group		Total	
	PHILOS	PRG		*p*-Value
Neer classification				
3				
Count	30	37	67	0.132
Percentage (%) within Neer classification	44.8%	55.2%	100.0%	
Percentage (%) within group	68.2%	84.1%	76.1%	
4				
Count	14	7	21	
Percentage (%) within Neer classification	66.7%	33.3%	100.0%	
Percentage (%) within group	31.8%	15.9%	23.9%	
Total				
Count	44	44	88	
Percentage (%) within Neer classification	50.0%	50.0%	100.0%	
Percentage (%) within group	100.0%	100.0%	100.0%	

### Functional outcomes

In the matched cohort, no statistically significant differences were found between PRG and PHILOS groups across all functional measures (Table 7): The effect sizes were small (0.14), and all mean differences remained below the MCID thresholds (e.g., DASH difference of 2.98 versus MCID of 10.2).

**Table 7:** Clinical results between groups

	Group	*N*	Mean	SD	*t* (df)	*p*-Value	Cohen’s *d*
OSS score	A: PGR	44	40.6591	10.36753	0.635 (86)	0.527	0.14
	B: PHILOS	44	39.2273	10.77651			
DASH score	A: PGR	44	15.2936	21.59903	−0.635 (86)	0.527	–0.14
	B: PHILOS	44	18.2765	22.45106			
SST score	A: PGR	44	10.1648	2.59188	0.635 (86)	0.527	0.14
	B: PHILOS	44	9.8068	2.69413			

*OSS* Oxford Shoulder Score, *DASH* Disabilities of the Arm, Shoulder and Hand questionnaire, *SST *Simple Shoulder Test, *t* *t*-test, *df * degrees of freedom

Mann–Whitney *U* tests were performed to compare functional outcomes between the treatment groups, with stratification by age. Among patients younger than 65 years (*n* = 30), no significant differences were observed between the PGR plate and PHILOS groups across all three outcome measures, including the OSS score (median: 47 versus 48; *p* = 0.552), DASH score (median: 2.083 versus 0.000; *p* = 0.552), and SST score (median: 11.75 versus 12; *p* = 0.552).

Similarly, in patients aged 65 years or older (*n* = 58), treatment groups showed comparable outcomes. The OSS scores were not significantly different between the PGR plate and PHILOS groups (median: 44 versus 39, *p* = 0.451). Similarly, the DASH scores (median: 8.333 versus 18.75, *p* = 0.451) and SST scores (median: 11 versus 9.75, *p* = 0.451) did not differ significantly between treatment groups in this older cohort. The results are presented in Table 7 and 8.

## Complications

The overall complication rate was 5.7%. Fisher’s exact test revealed no significant difference in the distribution of complications between the PGR plate and PHILOS groups (*p* = 0.167). Most patients had no complications (94.3% overall), with similar proportions in the PGR plate (97.7%) and PHILOS (90.9%) groups. Necrosis occurred infrequently (5.7% overall), without a statistically significant difference between groups (2.3% versus 9.1%). The results are presented in Table 9.

## Discussion

The aim of this retrospective study was to document the long-term outcomes of three- and four-part proximal humeral fractures that were stabilized with two different angular stabilization plates.

**Table 8:** Independent-samples Mann–Whitney *U* test *df* degrees of freedom

Age group	Group	*N*	Median	Interquartile range (IQR)	Standardized test statistics	*p*-Value
< 65 years						
OSS score	A: PGR	16	47	[43.500; 48.000]	0.667	0.552
	B: PHILOS	14	48	[45.000; 48.000]		
DASH score	A: PGR	16	2.083	[0.000; 9.375]	−0.667	0.552
	B: PHILOS	14	0.000	[0.000; 6.250]		
SST score	A: PGR	16	11.75	[10.875; 12.000]	0.667	0.552
	B: PHILOS	14	12	[11.250; 12.000]		
≥ 65 years						
OSS score	A: PGR	28	44	[33.750; 48.000]	−0.754	0.451
	B: PHILOS	30	39	[26.000; 47.250]		
DASH score	A: PGR	28	8.333	[0.000; 29.687]	0.754	0.451
	B: PHILOS	30	18.75	[1.563; 45.833]		
SST score	A: PGR	28	11	[8.436; 12.000]	−0.754	0.451
	B: PHILOS	30	9.75	[6.500; 11.812]		

When comparing our findings with other treatment strategies for three- and four-part proximal humeral fractures, the low complication rate observed in this series appears particularly noteworthy. While conservative treatment is often limited by residual stiffness and malunion in displaced fractures, and reverse shoulder arthroplasty, despite its reliability in elderly patients with poor bone quality, it carries risks of higher surgical invasiveness and concerns regarding long-term implant survival [3]

In this context, the favorable long-term outcomes and low complication profile observed in our ORIF cohort suggest that plate fixation remains a valid and less invasive option for selected patients when stable anatomic reduction can be achieved.

The findings indicated a low incidence of complications and satisfactory functional outcomes, as evaluated through three independent scoring systems, irrespective of the fixation plate design employed. Previous studies have reported variable complication rates following locking plate fixation for proximal humeral fractures, generally ranging from 15% to 35%, with avascular necrosis and loss of fixation being the most frequent concerns [1, 5, 8]. In contrast, our results revealed a markedly lower overall complication rate (5.7%), with no significant difference between the groups (Table 9).

**Table 9:** Fisher’s exact test for complications

	Group		Total	
	A: PGR	B: PHILOS		*p*-Value
Complications				
Absent				
Count	43	40	83	0.360
Percentage (%) within Neer classification	51.8%	48.2%	100.0%	
Percentage (%) within group	97.7%	90.9%	94.3%	
Necrosis				
Count	1	4	5	
Percentage (%) within Neer classification	20.0%	80.0%	100.0%	
Percentage (%) within group	2.3%	9.1%	5.7%	
Total				
Count	44	44	88	
Percentage (%) within Neer classification	50.0%	50.0%	100.0%	
Percentage (%) within group	100.0%	100.0%	100.0%	

Interestingly, no cases of nonunion, surgical site infection, or mechanical hardware failure—such as screw cutout or intraarticular migration—were recorded. While some studies on internal fixation for three- and four-part fractures report complication rates ranging from 15% to over 30%, our lower incidence may be related to the careful patient selection, specific surgical approach focused on preserving the medial soft-tissue envelope, and achieving stable anatomical reduction [5, 8]. Furthermore, the absence of documented shoulder stiffness or reoperations in this series might be partially explained by the long-term follow-up (mean 7 years), which may lead to a survival bias where patients with early catastrophic failures or poor outcomes may have undergone alternative treatments or revision procedures elsewhere. This aspect is now acknowledged as a potential limitation when interpreting the low complication rate observed.

The absence of statistically or clinically significant differences in the OSS, DASH, or SST score supports the hypothesis that implant geometry or design alone has a limited effect on long-term shoulder function, provided that anatomic reduction and stable fixation are achieved. Similar conclusions were drawn by Rollo et al. [11], who reported comparable short-term results between the same plate types, although our study extends this evidence to a follow-up duration exceeding one decade.

Interestingly, both implants maintained favorable results even in older patients (≥ 65 years), a subgroup often associated with osteoporotic bone and a higher risk of fixation failure. These findings suggest that both plate systems can offer reliable outcomes in geriatric populations when correct surgical principles are applied. However, despite the overall positive results, the small number of necrosis cases—mostly within the PHILOS group—warrants attention, aligning with prior literature linking greater screw density or medial calcar deficiency with humeral head ischemia [4, 8].

## Limitations

The present study has several limitations that should be considered when interpreting the results. First, its retrospective design may introduce inherent selection biases, although we performed propensity score matching (PSM) to mitigate differences in baseline characteristics such as age, follow-up duration, and fracture type. Despite these efforts, unmeasured confounding variables could still influence the functional outcomes and complication rates.

Second, the sample size, while sufficient to demonstrate clinical equivalence after matching, remains relatively small for detecting rare complications. This was particularly evident in our subgroup analyses by age, where the limited number of patients necessitated the use of nonparametric tests as normality could not be assumed.

A specific limitation concerns the classification of fracture displacement. In this study, we categorized fractures using the Neer classification system, which focuses on the number of displaced segments (three-part versus four-part). Consequently, we did not specifically subclassify fractures on the basis of the direction of displacement, such as varus or valgus patterns that may behave differently and carry a different risk profile for avascular necrosis.

While our groups were balanced regarding the severity of the fracture according to Neer, the potential influence of these specific displacement orientations on clinical and radiographic outcomes was not accounted for in our data.

Finally, the study utilized multiple functional scores (OSS, DASH, and SST), which showed a high degree of correlation. While this strengthens the consistency of our findings, future prospective trials with larger cohorts and more granular radiological assessment—including the medial hinge integrity and displacement direction—are needed to further validate the comparative efficacy of PRG and PHILOS systems.

Given the retrospective design and limited sample size, these results should be interpreted with caution. Further prospective, multicenter studies are warranted to confirm these findings and to better define the optimal indications for plate fixation in different patient populations.

## Conclusions

Both PGR and PHILOS locking plates provide durable fixation and comparable long-term functional recovery in the management of three- and four-part proximal humeral fractures in selected patients with complex proximal humeral fractures. Complication rates, including avascular necrosis and hardware-related events, were low and did not differ significantly between the two systems. When stable anatomic reduction and appropriate fixation principles are applied, both plate systems offer reliable long-term results.

## Data Availability

The datasets used and analyzed during the current study are available from the corresponding author on reasonable request.
